# 3β-Acet­oxy-12-oxoolean-28-oic acid benzyl ester

**DOI:** 10.1107/S1600536811010968

**Published:** 2011-04-07

**Authors:** Jun-yi Hu, Ying-qian Xu, Guo-bo Xu, Kang Wang, Peng Lei

**Affiliations:** a School of Chemical Engineering, University of Science and Technology Liaoning, Liaoning Anshan, 114051, People’s Republic of China; b Anshan Industry Research Institute, Liaoning Anshan, 114000, People’s Republic of China

## Abstract

The mol­ecule of title compound, C_39_H_56_O_5_, contains five fused six-membered rings, four of which (rings *A*, *B*, *D* and *E*) adopt a chair conformation, while the other (ring *C*) has a half chair conformation. The acet­oxy and carb­oxy­benzyl groups occupy equatorial positions.

## Related literature

For oleanolic acid derivatives, see: Honda *et al.* (2003 ▶); Liu (1995 ▶); Matsuda *et al.* (1999 ▶); Sun *et al.* (2003 ▶). For standard bond lengths, see: Allen *et al.* (1987 ▶). 
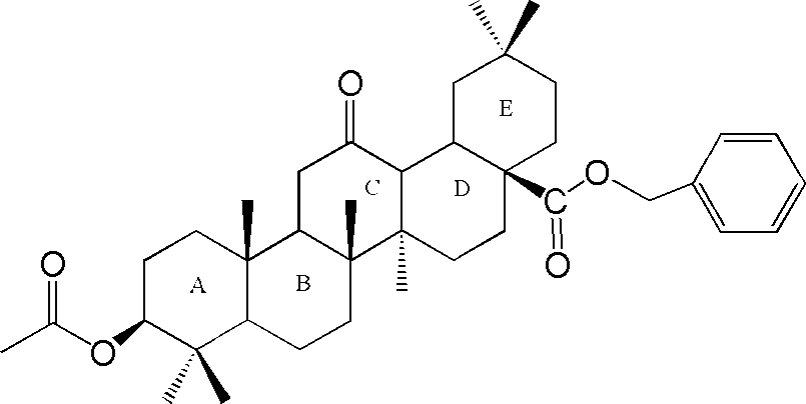


## Experimental

### 

#### Crystal data

C_39_H_56_O_5_
*M* *_r_* = 604.84
Orthorhombic, 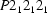

*a* = 6.9775 (3) Å
*b* = 12.4235 (5) Å
*c* = 39.1480 (16) Å
*V* = 3393.5 (2) Å^3^
*Z* = 4
Mo *K*α radiation
μ = 0.08 mm^−1^
*T* = 113 K
0.16 × 0.14 × 0.12 mm

#### Data collection

Rigaku Saturn diffractometer
Absorption correction: multi-scan (*CrystalClear*;  Rigaku/MSC, 2005 ▶)*T*_min_ = 0.980, *T*_max_ = 0.991
31130 measured reflections
4606 independent reflections
4201 reflections with *I* > 2σ(*I*)
*R*_int_ = 0.061

#### Refinement

*R*[*F* ^2^ > 2σ(*F* ^2^)] = 0.050
*wR*(*F* ^2^) = 0.114
*S* = 1.09
4606 reflections
406 parameters
H-atom parameters constrained
Δρ_max_ = 0.24 e Å^−3^
Δρ_min_ = −0.18 e Å^−3^

### 

Data collection: *CrystalClear* (Rigaku/MSC, 2005 ▶); cell refinement: *CrystalClear*; data reduction: *CrystalClear*; program(s) used to solve structure: *SHELXS97* (Sheldrick, 2008 ▶); program(s) used to refine structure: *SHELXL97* (Sheldrick, 2008 ▶); molecular graphics: *SHELXTL* (Sheldrick, 2008 ▶); software used to prepare material for publication: *SHELXTL*.

## Supplementary Material

Crystal structure: contains datablocks global, I. DOI: 10.1107/S1600536811010968/yk2003sup1.cif

Structure factors: contains datablocks I. DOI: 10.1107/S1600536811010968/yk2003Isup2.hkl

Additional supplementary materials:  crystallographic information; 3D view; checkCIF report

## Figures and Tables

**Fig. 1.**
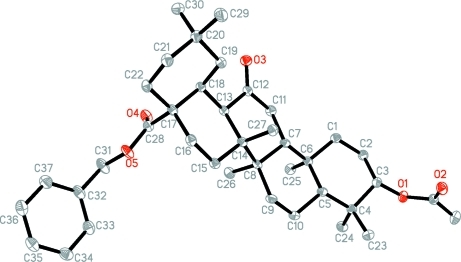
View of the molecule of C39H56O5 showing the atom-labeling scheme. Displacement ellipsoids are drawn at the 35% probability level. H atoms are omitted for clarity.

## supplementary crystallographic information

### Comment

Oleanolic acid derivatives are triterpenoid compounds that exist widely in food, medicinal herbs and other plants (Liu, 1995; Honda *et al.*, 2003; Matsuda *et al.*, 1999). Recently, our laboratories have disclosed the design and synthesis of a novel series of ET_A_ receptor selective antagonists, based on oleanolic acid derivatives. Herewith, we report the synthesis and crystal structure of the title compound.

In the molecule of the title compound (Fig. 1) bond lengths and angles are generally within normal ranges (Allen *et al.*, 1987). It contains five cyclohexane rings [A(C1—C6), B(C5—C10), C(C7,C8,C11—C14), D(C13—C18) and E(C17—C22)]. The ring A, B, D and E take a chair conformation, and the ring C has a half-chair conformation, in which the carboxybenzyl and acetoxy groups occupy the equatorial positions.

### Experimental

Commercially available oleanolic acid, benzyl bromide and K_2_CO_3_ in dry tetrahydrofuran was stirred at room temperature overnight to give benzyl ester in 91% yield. Reaction of benzyl ester with acetic anhydride in dry pyridine gave acetyl oleanolic acid benzyl ester in 96% yield. Treatment of acetyl oleanolic acid benzyl ester with 1,4-dioxane-acetic acid mixture, followed by adding the solution of H_2_O_2_ in acetic acid, afforded the title compound (yield 64% after column chromatography purification). Crystals suitable for X-ray structure analysis were obtained by slow evaporation of a solution in methanol at room temperature.

### Refinement

The absolute configuration could not be established because of the absence of significant anomalous effects and has been assigned arbitrary. Friedel pairs were merged. H atoms were positioned geometrically and allowed to ride on their parent atoms with C—H = 0.95–1.00 Å and *U*_iso_(H) =1.2*U*_eq_(parent) or 1.5*U*_eq_(parent).

### Crystal data

**Table:**

C_39_H_56_O_5_	*F*(000) = 1320
*M**_r_* = 604.84	*D*_x_ = 1.184 Mg m^−^^3^
Orthorhombic, *P*2_1_2_1_2_1_	Mo *K*α radiation, λ = 0.71073 Å
Hall symbol: P 2ac 2ab	Cell parameters from 6474 reflections
*a* = 6.9775 (3) Å	θ = 1.7–27.9°
*b* = 12.4235 (5) Å	µ = 0.08 mm^−^^1^
*c* = 39.1480 (16) Å	*T* = 113 K
*V* = 3393.5 (2) Å^3^	Block, colourless
*Z* = 4	0.16 × 0.14 × 0.12 mm

### Data collection

**Table:**

Rigaku Saturn diffractometer	4606 independent reflections
Radiation source: rotating anode	4201 reflections with *I* > 2σ(*I*)
confocal	*R*_int_ = 0.061
Detector resolution: 7.31 pixels mm^-1^	θ_max_ = 27.9°, θ_min_ = 1.7°
ω scans	*h* = −8→9
Absorption correction: multi-scan *CrystalClear*	*k* = −16→16
*T*_min_ = 0.980, *T*_max_ = 0.991	*l* = −48→51
31130 measured reflections	

### Refinement

**Table:**

Refinement on *F*^2^	Secondary atom site location: difference Fourier map
Least-squares matrix: full	Hydrogen site location: inferred from neighbouring sites
*R*[*F*^2^ > 2σ(*F*^2^)] = 0.050	H-atom parameters constrained
*wR*(*F*^2^) = 0.114	*w* = 1/[σ^2^(*F*_o_^2^) + (0.0532*P*)^2^ + 0.4893*P*] where *P* = (*F*_o_^2^ + 2*F*_c_^2^)/3
*S* = 1.09	(Δ/σ)_max_ = 0.001
4606 reflections	Δρ_max_ = 0.24 e Å^−^^3^
406 parameters	Δρ_min_ = −0.18 e Å^−^^3^
0 restraints	Extinction correction: *SHELXL*, Fc^*^=kFc[1+0.001xFc^2^λ^3^/sin(2θ)]^-1/4^
Primary atom site location: structure-invariant direct methods	Extinction coefficient: 0.0053 (7)

### Special details

**Table:**

Geometry. All e.s.d.'s (except the e.s.d. in the dihedral angle between two l.s. planes) are estimated using the full covariance matrix. The cell e.s.d.'s are taken into account individually in the estimation of e.s.d.'s in distances, angles and torsion angles; correlations between e.s.d.'s in cell parameters are only used when they are defined by crystal symmetry. An approximate (isotropic) treatment of cell e.s.d.'s is used for estimating e.s.d.'s involving l.s. planes.
Refinement. Refinement of *F*^2^ against ALL reflections. The weighted *R*-factor *wR* and goodness of fit *S* are based on *F*^2^, conventional *R*-factors *R* are based on *F*, with *F* set to zero for negative *F*^2^. The threshold expression of *F*^2^ > σ(*F*^2^) is used only for calculating *R*-factors(gt) *etc*. and is not relevant to the choice of reflections for refinement. *R*-factors based on *F*^2^ are statistically about twice as large as those based on *F*, and *R*- factors based on ALL data will be even larger.

### Fractional atomic coordinates and isotropic or equivalent isotropic displacement parameters (Å^2^)

**Table:**

	*x*	*y*	*z*	*U*_iso_*/*U*_eq_	
O1	0.9465 (2)	0.71768 (14)	0.64283 (4)	0.0274 (4)	
O2	1.2672 (3)	0.74643 (14)	0.64732 (5)	0.0335 (4)	
O3	0.6349 (3)	0.89260 (13)	0.85384 (4)	0.0277 (4)	
O4	0.3626 (3)	0.60770 (14)	0.90948 (5)	0.0315 (4)	
O5	0.5121 (3)	0.45703 (13)	0.92650 (5)	0.0314 (4)	
C1	0.7536 (4)	0.80684 (18)	0.72827 (6)	0.0250 (5)	
H1A	0.6580	0.8629	0.7340	0.030*	
H1B	0.8770	0.8280	0.7388	0.030*	
C2	0.7781 (4)	0.80269 (19)	0.68926 (6)	0.0267 (5)	
H2A	0.6533	0.7870	0.6783	0.032*	
H2B	0.8236	0.8733	0.6808	0.032*	
C3	0.9216 (4)	0.71608 (18)	0.67993 (5)	0.0242 (5)	
H3	1.0471	0.7333	0.6910	0.029*	
C4	0.8594 (4)	0.60222 (18)	0.69087 (6)	0.0240 (5)	
C5	0.8254 (3)	0.60835 (17)	0.73047 (6)	0.0224 (5)	
H5	0.9535	0.6277	0.7401	0.027*	
C6	0.6882 (3)	0.69845 (18)	0.74376 (6)	0.0217 (5)	
C7	0.7249 (3)	0.70419 (16)	0.78349 (5)	0.0211 (5)	
H7	0.8628	0.7250	0.7856	0.025*	
C8	0.7082 (3)	0.59695 (17)	0.80415 (6)	0.0209 (5)	
C9	0.8147 (4)	0.50465 (17)	0.78578 (6)	0.0257 (5)	
H9A	0.9541	0.5134	0.7895	0.031*	
H9B	0.7757	0.4353	0.7961	0.031*	
C10	0.7762 (4)	0.50003 (17)	0.74724 (6)	0.0260 (5)	
H10A	0.6396	0.4829	0.7432	0.031*	
H10B	0.8547	0.4423	0.7368	0.031*	
C11	0.6151 (4)	0.79569 (18)	0.80144 (6)	0.0250 (5)	
H11A	0.6530	0.8647	0.7908	0.030*	
H11B	0.4765	0.7855	0.7971	0.030*	
C12	0.6455 (3)	0.80507 (17)	0.83995 (6)	0.0216 (5)	
C13	0.6707 (3)	0.70130 (17)	0.85983 (5)	0.0202 (5)	
H13	0.5409	0.6672	0.8594	0.024*	
C14	0.8015 (3)	0.61930 (17)	0.84082 (6)	0.0209 (5)	
C15	0.8127 (4)	0.51426 (17)	0.86228 (6)	0.0236 (5)	
H15A	0.6891	0.4756	0.8603	0.028*	
H15B	0.9136	0.4674	0.8526	0.028*	
C16	0.8564 (4)	0.53346 (18)	0.90039 (6)	0.0246 (5)	
H16A	0.8545	0.4636	0.9126	0.030*	
H16B	0.9867	0.5641	0.9026	0.030*	
C17	0.7116 (3)	0.61004 (18)	0.91721 (6)	0.0222 (5)	
C18	0.7123 (3)	0.71981 (17)	0.89836 (5)	0.0204 (5)	
H18	0.6031	0.7628	0.9077	0.025*	
C19	0.8967 (4)	0.78302 (18)	0.90615 (6)	0.0239 (5)	
H19A	0.8898	0.8535	0.8944	0.029*	
H19B	1.0070	0.7432	0.8965	0.029*	
C20	0.9346 (4)	0.8027 (2)	0.94448 (6)	0.0275 (5)	
C21	0.9352 (4)	0.6923 (2)	0.96255 (6)	0.0286 (5)	
H21A	1.0479	0.6506	0.9547	0.034*	
H21B	0.9485	0.7037	0.9875	0.034*	
C22	0.7538 (4)	0.62654 (18)	0.95579 (6)	0.0262 (5)	
H22A	0.6433	0.6635	0.9665	0.031*	
H22B	0.7672	0.5552	0.9668	0.031*	
C23	1.0282 (4)	0.52563 (19)	0.68376 (6)	0.0310 (6)	
H23A	1.0650	0.5309	0.6597	0.046*	
H23B	0.9897	0.4515	0.6889	0.046*	
H23C	1.1374	0.5457	0.6982	0.046*	
C24	0.6862 (4)	0.5619 (2)	0.67035 (6)	0.0311 (6)	
H24A	0.5830	0.6153	0.6715	0.047*	
H24B	0.6413	0.4936	0.6800	0.047*	
H24C	0.7237	0.5511	0.6465	0.047*	
C25	0.4751 (4)	0.6817 (2)	0.73452 (6)	0.0286 (5)	
H25A	0.4432	0.6051	0.7363	0.043*	
H25B	0.4522	0.7065	0.7111	0.043*	
H25C	0.3946	0.7229	0.7503	0.043*	
C26	0.4960 (4)	0.56045 (19)	0.80771 (6)	0.0276 (5)	
H26A	0.4524	0.5294	0.7860	0.041*	
H26B	0.4160	0.6226	0.8135	0.041*	
H26C	0.4860	0.5062	0.8258	0.041*	
C27	1.0085 (3)	0.66348 (18)	0.83667 (6)	0.0232 (5)	
H27A	1.0857	0.6423	0.8565	0.035*	
H27B	1.0046	0.7422	0.8351	0.035*	
H27C	1.0658	0.6338	0.8158	0.035*	
C28	0.5095 (4)	0.56109 (18)	0.91635 (6)	0.0252 (5)	
C29	1.1334 (4)	0.8542 (2)	0.94813 (7)	0.0374 (7)	
H29A	1.1345	0.9240	0.9365	0.056*	
H29B	1.2300	0.8070	0.9378	0.056*	
H29C	1.1626	0.8646	0.9724	0.056*	
C30	0.7847 (4)	0.8789 (2)	0.96021 (6)	0.0360 (6)	
H30A	0.8141	0.8902	0.9844	0.054*	
H30B	0.6569	0.8469	0.9580	0.054*	
H30C	0.7877	0.9481	0.9482	0.054*	
C31	0.3242 (4)	0.4050 (2)	0.92880 (8)	0.0384 (7)	
H31A	0.2654	0.3995	0.9059	0.046*	
H31B	0.2378	0.4475	0.9437	0.046*	
C32	0.3546 (4)	0.29481 (19)	0.94361 (7)	0.0304 (6)	
C33	0.3912 (4)	0.2077 (2)	0.92240 (7)	0.0326 (6)	
H33	0.3971	0.2183	0.8984	0.039*	
C34	0.4195 (4)	0.1051 (2)	0.93572 (7)	0.0350 (6)	
H34	0.4449	0.0461	0.9210	0.042*	
C35	0.4104 (4)	0.0901 (2)	0.97059 (7)	0.0383 (7)	
H35	0.4310	0.0204	0.9799	0.046*	
C36	0.3715 (5)	0.1755 (2)	0.99212 (7)	0.0436 (7)	
H36	0.3623	0.1642	1.0161	0.052*	
C37	0.3456 (4)	0.2783 (2)	0.97862 (7)	0.0394 (7)	
H37	0.3218	0.3373	0.9935	0.047*	
C38	1.1264 (4)	0.72903 (19)	0.63014 (6)	0.0276 (5)	
C39	1.1258 (4)	0.7154 (2)	0.59200 (6)	0.0372 (6)	
H39A	1.2318	0.7566	0.5821	0.056*	
H39B	1.0040	0.7416	0.5827	0.056*	
H39C	1.1412	0.6390	0.5864	0.056*	

### Atomic displacement parameters (Å^2^)

**Table:**

	*U*^11^	*U*^22^	*U*^33^	*U*^12^	*U*^13^	*U*^23^
O1	0.0211 (9)	0.0358 (9)	0.0254 (8)	−0.0005 (7)	0.0013 (7)	−0.0001 (7)
O2	0.0235 (10)	0.0373 (10)	0.0396 (10)	−0.0029 (8)	0.0000 (8)	0.0018 (8)
O3	0.0323 (10)	0.0215 (7)	0.0294 (8)	0.0048 (8)	−0.0013 (8)	0.0008 (7)
O4	0.0194 (9)	0.0304 (9)	0.0448 (10)	0.0015 (8)	0.0003 (8)	0.0097 (8)
O5	0.0194 (10)	0.0228 (8)	0.0519 (11)	−0.0029 (7)	−0.0005 (8)	0.0109 (8)
C1	0.0280 (13)	0.0216 (11)	0.0253 (11)	0.0038 (10)	−0.0015 (10)	0.0005 (9)
C2	0.0298 (14)	0.0257 (11)	0.0246 (11)	0.0007 (11)	−0.0001 (10)	0.0022 (9)
C3	0.0230 (13)	0.0275 (11)	0.0222 (11)	−0.0019 (10)	−0.0010 (9)	−0.0012 (9)
C4	0.0228 (12)	0.0238 (10)	0.0254 (11)	−0.0008 (10)	−0.0001 (10)	−0.0032 (9)
C5	0.0221 (13)	0.0194 (10)	0.0259 (11)	0.0016 (10)	−0.0034 (10)	−0.0003 (9)
C6	0.0213 (12)	0.0193 (10)	0.0246 (11)	0.0010 (9)	−0.0013 (9)	0.0008 (9)
C7	0.0212 (12)	0.0160 (9)	0.0260 (11)	0.0025 (9)	−0.0020 (9)	0.0008 (8)
C8	0.0189 (12)	0.0174 (9)	0.0265 (11)	−0.0015 (9)	−0.0012 (9)	0.0017 (8)
C9	0.0333 (14)	0.0162 (10)	0.0278 (12)	0.0015 (10)	−0.0020 (11)	−0.0004 (9)
C10	0.0293 (14)	0.0193 (10)	0.0294 (12)	−0.0002 (10)	−0.0007 (11)	−0.0031 (9)
C11	0.0245 (13)	0.0238 (11)	0.0267 (11)	0.0047 (10)	−0.0031 (10)	0.0042 (9)
C12	0.0173 (12)	0.0202 (10)	0.0273 (11)	0.0023 (9)	0.0002 (9)	0.0036 (9)
C13	0.0183 (12)	0.0187 (9)	0.0237 (10)	0.0007 (9)	−0.0012 (9)	0.0019 (8)
C14	0.0186 (12)	0.0182 (10)	0.0259 (11)	−0.0001 (9)	−0.0015 (9)	0.0017 (8)
C15	0.0228 (13)	0.0197 (10)	0.0283 (12)	0.0028 (9)	0.0005 (10)	0.0030 (9)
C16	0.0219 (13)	0.0234 (11)	0.0285 (12)	0.0022 (10)	−0.0002 (10)	0.0056 (9)
C17	0.0182 (12)	0.0210 (10)	0.0276 (11)	−0.0015 (9)	−0.0017 (9)	0.0033 (9)
C18	0.0194 (12)	0.0182 (10)	0.0237 (10)	0.0016 (9)	0.0004 (9)	0.0021 (8)
C19	0.0244 (13)	0.0226 (11)	0.0248 (11)	−0.0009 (10)	−0.0001 (10)	0.0024 (9)
C20	0.0267 (14)	0.0311 (12)	0.0246 (11)	−0.0018 (11)	−0.0016 (10)	0.0011 (10)
C21	0.0277 (14)	0.0337 (13)	0.0245 (11)	−0.0022 (11)	−0.0042 (10)	0.0027 (10)
C22	0.0279 (14)	0.0265 (11)	0.0241 (11)	−0.0008 (10)	−0.0005 (10)	0.0061 (9)
C23	0.0331 (15)	0.0277 (12)	0.0321 (13)	0.0029 (11)	0.0023 (11)	−0.0036 (10)
C24	0.0301 (15)	0.0324 (12)	0.0308 (13)	−0.0079 (11)	−0.0045 (11)	−0.0049 (10)
C25	0.0240 (13)	0.0334 (12)	0.0283 (12)	0.0022 (11)	−0.0040 (10)	0.0003 (10)
C26	0.0216 (13)	0.0292 (12)	0.0320 (12)	−0.0039 (11)	−0.0038 (11)	0.0020 (10)
C27	0.0180 (12)	0.0249 (10)	0.0267 (11)	0.0007 (10)	0.0002 (10)	0.0017 (9)
C28	0.0242 (14)	0.0247 (11)	0.0269 (12)	−0.0003 (10)	0.0002 (10)	0.0026 (9)
C29	0.0408 (17)	0.0415 (14)	0.0300 (13)	−0.0147 (13)	−0.0062 (13)	0.0002 (11)
C30	0.0464 (18)	0.0291 (12)	0.0324 (13)	−0.0015 (13)	0.0032 (13)	−0.0028 (10)
C31	0.0182 (14)	0.0337 (13)	0.0634 (18)	−0.0061 (12)	−0.0007 (13)	0.0156 (13)
C32	0.0164 (13)	0.0277 (12)	0.0471 (14)	−0.0044 (11)	−0.0001 (11)	0.0088 (11)
C33	0.0228 (14)	0.0357 (13)	0.0394 (14)	−0.0028 (11)	0.0000 (11)	0.0079 (12)
C34	0.0292 (15)	0.0295 (12)	0.0462 (15)	−0.0054 (12)	−0.0002 (12)	−0.0010 (11)
C35	0.0344 (17)	0.0305 (13)	0.0500 (17)	−0.0054 (12)	−0.0004 (13)	0.0100 (12)
C36	0.052 (2)	0.0412 (15)	0.0375 (14)	−0.0041 (15)	0.0039 (14)	0.0095 (12)
C37	0.0404 (18)	0.0333 (13)	0.0447 (15)	−0.0041 (13)	0.0060 (13)	0.0006 (12)
C38	0.0220 (13)	0.0239 (11)	0.0370 (13)	0.0023 (10)	0.0025 (11)	0.0024 (10)
C39	0.0291 (15)	0.0497 (16)	0.0329 (13)	0.0043 (13)	0.0057 (12)	0.0002 (12)

### Geometric parameters (Å, °)

**Table:**

O1—C38	1.357 (3)	C18—C19	1.538 (3)
O1—C3	1.463 (3)	C18—H18	1.0000
O2—C38	1.210 (3)	C19—C20	1.543 (3)
O3—C12	1.218 (3)	C19—H19A	0.9900
O4—C28	1.208 (3)	C19—H19B	0.9900
O5—C28	1.353 (3)	C20—C29	1.535 (4)
O5—C31	1.465 (3)	C20—C30	1.539 (4)
C1—C2	1.537 (3)	C20—C21	1.543 (3)
C1—C6	1.546 (3)	C21—C22	1.529 (3)
C1—H1A	0.9900	C21—H21A	0.9900
C1—H1B	0.9900	C21—H21B	0.9900
C2—C3	1.514 (3)	C22—H22A	0.9900
C2—H2A	0.9900	C22—H22B	0.9900
C2—H2B	0.9900	C23—H23A	0.9800
C3—C4	1.540 (3)	C23—H23B	0.9800
C3—H3	1.0000	C23—H23C	0.9800
C4—C24	1.536 (3)	C24—H24A	0.9800
C4—C23	1.540 (3)	C24—H24B	0.9800
C4—C5	1.570 (3)	C24—H24C	0.9800
C5—C10	1.536 (3)	C25—H25A	0.9800
C5—C6	1.562 (3)	C25—H25B	0.9800
C5—H5	1.0000	C25—H25C	0.9800
C6—C25	1.545 (3)	C26—H26A	0.9800
C6—C7	1.578 (3)	C26—H26B	0.9800
C7—C11	1.540 (3)	C26—H26C	0.9800
C7—C8	1.563 (3)	C27—H27A	0.9800
C7—H7	1.0000	C27—H27B	0.9800
C8—C9	1.544 (3)	C27—H27C	0.9800
C8—C26	1.554 (3)	C29—H29A	0.9800
C8—C14	1.601 (3)	C29—H29B	0.9800
C9—C10	1.533 (3)	C29—H29C	0.9800
C9—H9A	0.9900	C30—H30A	0.9800
C9—H9B	0.9900	C30—H30B	0.9800
C10—H10A	0.9900	C30—H30C	0.9800
C10—H10B	0.9900	C31—C32	1.502 (3)
C11—C12	1.527 (3)	C31—H31A	0.9900
C11—H11A	0.9900	C31—H31B	0.9900
C11—H11B	0.9900	C32—C37	1.387 (4)
C12—C13	1.516 (3)	C32—C33	1.388 (4)
C13—C18	1.553 (3)	C33—C34	1.391 (4)
C13—C14	1.557 (3)	C33—H33	0.9500
C13—H13	1.0000	C34—C35	1.379 (4)
C14—C27	1.554 (3)	C34—H34	0.9500
C14—C15	1.554 (3)	C35—C36	1.382 (4)
C15—C16	1.542 (3)	C35—H35	0.9500
C15—H15A	0.9900	C36—C37	1.394 (4)
C15—H15B	0.9900	C36—H36	0.9500
C16—C17	1.536 (3)	C37—H37	0.9500
C16—H16A	0.9900	C38—C39	1.503 (3)
C16—H16B	0.9900	C39—H39A	0.9800
C17—C28	1.535 (3)	C39—H39B	0.9800
C17—C18	1.551 (3)	C39—H39C	0.9800
C17—C22	1.552 (3)		
			
C38—O1—C3	118.35 (19)	C17—C18—H18	107.0
C28—O5—C31	115.34 (19)	C13—C18—H18	107.0
C2—C1—C6	113.16 (18)	C18—C19—C20	114.63 (19)
C2—C1—H1A	108.9	C18—C19—H19A	108.6
C6—C1—H1A	108.9	C20—C19—H19A	108.6
C2—C1—H1B	108.9	C18—C19—H19B	108.6
C6—C1—H1B	108.9	C20—C19—H19B	108.6
H1A—C1—H1B	107.8	H19A—C19—H19B	107.6
C3—C2—C1	109.71 (19)	C29—C20—C30	108.7 (2)
C3—C2—H2A	109.7	C29—C20—C19	108.1 (2)
C1—C2—H2A	109.7	C30—C20—C19	111.7 (2)
C3—C2—H2B	109.7	C29—C20—C21	109.0 (2)
C1—C2—H2B	109.7	C30—C20—C21	111.4 (2)
H2A—C2—H2B	108.2	C19—C20—C21	107.8 (2)
O1—C3—C2	107.98 (18)	C22—C21—C20	113.1 (2)
O1—C3—C4	108.78 (18)	C22—C21—H21A	109.0
C2—C3—C4	113.5 (2)	C20—C21—H21A	109.0
O1—C3—H3	108.8	C22—C21—H21B	109.0
C2—C3—H3	108.8	C20—C21—H21B	109.0
C4—C3—H3	108.8	H21A—C21—H21B	107.8
C24—C4—C23	107.83 (19)	C21—C22—C17	113.34 (19)
C24—C4—C3	112.1 (2)	C21—C22—H22A	108.9
C23—C4—C3	107.6 (2)	C17—C22—H22A	108.9
C24—C4—C5	114.4 (2)	C21—C22—H22B	108.9
C23—C4—C5	108.91 (19)	C17—C22—H22B	108.9
C3—C4—C5	105.82 (17)	H22A—C22—H22B	107.7
C10—C5—C6	110.40 (19)	C4—C23—H23A	109.5
C10—C5—C4	114.42 (18)	C4—C23—H23B	109.5
C6—C5—C4	117.15 (18)	H23A—C23—H23B	109.5
C10—C5—H5	104.4	C4—C23—H23C	109.5
C6—C5—H5	104.4	H23A—C23—H23C	109.5
C4—C5—H5	104.4	H23B—C23—H23C	109.5
C25—C6—C1	108.03 (19)	C4—C24—H24A	109.5
C25—C6—C5	114.53 (19)	C4—C24—H24B	109.5
C1—C6—C5	108.23 (19)	H24A—C24—H24B	109.5
C25—C6—C7	113.15 (19)	C4—C24—H24C	109.5
C1—C6—C7	107.43 (17)	H24A—C24—H24C	109.5
C5—C6—C7	105.16 (17)	H24B—C24—H24C	109.5
C11—C7—C8	110.84 (18)	C6—C25—H25A	109.5
C11—C7—C6	113.74 (18)	C6—C25—H25B	109.5
C8—C7—C6	117.36 (17)	H25A—C25—H25B	109.5
C11—C7—H7	104.5	C6—C25—H25C	109.5
C8—C7—H7	104.5	H25A—C25—H25C	109.5
C6—C7—H7	104.5	H25B—C25—H25C	109.5
C9—C8—C26	106.47 (19)	C8—C26—H26A	109.5
C9—C8—C7	110.85 (18)	C8—C26—H26B	109.5
C26—C8—C7	111.48 (19)	H26A—C26—H26B	109.5
C9—C8—C14	110.53 (18)	C8—C26—H26C	109.5
C26—C8—C14	110.96 (19)	H26A—C26—H26C	109.5
C7—C8—C14	106.62 (17)	H26B—C26—H26C	109.5
C10—C9—C8	113.69 (19)	C14—C27—H27A	109.5
C10—C9—H9A	108.8	C14—C27—H27B	109.5
C8—C9—H9A	108.8	H27A—C27—H27B	109.5
C10—C9—H9B	108.8	C14—C27—H27C	109.5
C8—C9—H9B	108.8	H27A—C27—H27C	109.5
H9A—C9—H9B	107.7	H27B—C27—H27C	109.5
C9—C10—C5	110.40 (18)	O4—C28—O5	122.3 (2)
C9—C10—H10A	109.6	O4—C28—C17	126.5 (2)
C5—C10—H10A	109.6	O5—C28—C17	111.1 (2)
C9—C10—H10B	109.6	C20—C29—H29A	109.5
C5—C10—H10B	109.6	C20—C29—H29B	109.5
H10A—C10—H10B	108.1	H29A—C29—H29B	109.5
C12—C11—C7	115.97 (18)	C20—C29—H29C	109.5
C12—C11—H11A	108.3	H29A—C29—H29C	109.5
C7—C11—H11A	108.3	H29B—C29—H29C	109.5
C12—C11—H11B	108.3	C20—C30—H30A	109.5
C7—C11—H11B	108.3	C20—C30—H30B	109.5
H11A—C11—H11B	107.4	H30A—C30—H30B	109.5
O3—C12—C13	122.5 (2)	C20—C30—H30C	109.5
O3—C12—C11	120.04 (19)	H30A—C30—H30C	109.5
C13—C12—C11	117.27 (19)	H30B—C30—H30C	109.5
C12—C13—C18	113.23 (17)	O5—C31—C32	107.4 (2)
C12—C13—C14	112.28 (18)	O5—C31—H31A	110.2
C18—C13—C14	116.85 (18)	C32—C31—H31A	110.2
C12—C13—H13	104.3	O5—C31—H31B	110.2
C18—C13—H13	104.3	C32—C31—H31B	110.2
C14—C13—H13	104.3	H31A—C31—H31B	108.5
C27—C14—C15	107.85 (18)	C37—C32—C33	119.0 (2)
C27—C14—C13	111.32 (18)	C37—C32—C31	120.7 (3)
C15—C14—C13	108.69 (18)	C33—C32—C31	120.4 (2)
C27—C14—C8	110.22 (18)	C32—C33—C34	121.1 (2)
C15—C14—C8	111.08 (17)	C32—C33—H33	119.5
C13—C14—C8	107.69 (18)	C34—C33—H33	119.5
C16—C15—C14	113.79 (18)	C35—C34—C33	119.2 (3)
C16—C15—H15A	108.8	C35—C34—H34	120.4
C14—C15—H15A	108.8	C33—C34—H34	120.4
C16—C15—H15B	108.8	C34—C35—C36	120.6 (3)
C14—C15—H15B	108.8	C34—C35—H35	119.7
H15A—C15—H15B	107.7	C36—C35—H35	119.7
C17—C16—C15	112.36 (19)	C35—C36—C37	119.8 (3)
C17—C16—H16A	109.1	C35—C36—H36	120.1
C15—C16—H16A	109.1	C37—C36—H36	120.1
C17—C16—H16B	109.1	C32—C37—C36	120.3 (3)
C15—C16—H16B	109.1	C32—C37—H37	119.8
H16A—C16—H16B	107.9	C36—C37—H37	119.8
C28—C17—C16	110.44 (19)	O2—C38—O1	124.5 (2)
C28—C17—C18	109.94 (18)	O2—C38—C39	125.1 (2)
C16—C17—C18	109.78 (18)	O1—C38—C39	110.4 (2)
C28—C17—C22	104.36 (19)	C38—C39—H39A	109.5
C16—C17—C22	111.95 (19)	C38—C39—H39B	109.5
C18—C17—C22	110.26 (18)	H39A—C39—H39B	109.5
C19—C18—C17	110.94 (18)	C38—C39—H39C	109.5
C19—C18—C13	115.12 (18)	H39A—C39—H39C	109.5
C17—C18—C13	109.36 (17)	H39B—C39—H39C	109.5
C19—C18—H18	107.0		
			
C6—C1—C2—C3	−57.7 (3)	C26—C8—C14—C27	−175.96 (17)
C38—O1—C3—C2	124.9 (2)	C7—C8—C14—C27	−54.4 (2)
C38—O1—C3—C4	−111.5 (2)	C9—C8—C14—C15	−53.3 (2)
C1—C2—C3—O1	−177.77 (19)	C26—C8—C14—C15	64.6 (2)
C1—C2—C3—C4	61.5 (3)	C7—C8—C14—C15	−173.87 (19)
O1—C3—C4—C24	−51.8 (3)	C9—C8—C14—C13	−172.22 (18)
C2—C3—C4—C24	68.4 (3)	C26—C8—C14—C13	−54.3 (2)
O1—C3—C4—C23	66.5 (2)	C7—C8—C14—C13	67.2 (2)
C2—C3—C4—C23	−173.21 (19)	C27—C14—C15—C16	72.6 (2)
O1—C3—C4—C5	−177.16 (18)	C13—C14—C15—C16	−48.2 (3)
C2—C3—C4—C5	−56.9 (2)	C8—C14—C15—C16	−166.6 (2)
C24—C4—C5—C10	60.5 (3)	C14—C15—C16—C17	55.9 (3)
C23—C4—C5—C10	−60.3 (3)	C15—C16—C17—C28	62.7 (2)
C3—C4—C5—C10	−175.7 (2)	C15—C16—C17—C18	−58.7 (3)
C24—C4—C5—C6	−71.1 (3)	C15—C16—C17—C22	178.55 (19)
C23—C4—C5—C6	168.2 (2)	C28—C17—C18—C19	166.22 (19)
C3—C4—C5—C6	52.8 (3)	C16—C17—C18—C19	−72.1 (2)
C2—C1—C6—C25	−73.3 (2)	C22—C17—C18—C19	51.7 (3)
C2—C1—C6—C5	51.3 (3)	C28—C17—C18—C13	−65.7 (2)
C2—C1—C6—C7	164.3 (2)	C16—C17—C18—C13	55.9 (3)
C10—C5—C6—C25	−63.7 (2)	C22—C17—C18—C13	179.73 (19)
C4—C5—C6—C25	69.6 (3)	C12—C13—C18—C19	−61.1 (3)
C10—C5—C6—C1	175.72 (18)	C14—C13—C18—C19	71.7 (2)
C4—C5—C6—C1	−50.9 (3)	C12—C13—C18—C17	173.25 (19)
C10—C5—C6—C7	61.1 (2)	C14—C13—C18—C17	−53.9 (3)
C4—C5—C6—C7	−165.52 (19)	C17—C18—C19—C20	−56.2 (3)
C25—C6—C7—C11	−59.2 (3)	C13—C18—C19—C20	178.95 (19)
C1—C6—C7—C11	59.9 (3)	C18—C19—C20—C29	173.4 (2)
C5—C6—C7—C11	175.05 (19)	C18—C19—C20—C30	−67.1 (3)
C25—C6—C7—C8	72.5 (3)	C18—C19—C20—C21	55.7 (3)
C1—C6—C7—C8	−168.4 (2)	C29—C20—C21—C22	−171.2 (2)
C5—C6—C7—C8	−53.3 (3)	C30—C20—C21—C22	68.9 (3)
C11—C7—C8—C9	178.83 (19)	C19—C20—C21—C22	−54.0 (3)
C6—C7—C8—C9	45.8 (3)	C20—C21—C22—C17	54.9 (3)
C11—C7—C8—C26	60.4 (2)	C28—C17—C22—C21	−170.27 (19)
C6—C7—C8—C26	−72.6 (3)	C16—C17—C22—C21	70.3 (2)
C11—C7—C8—C14	−60.8 (2)	C18—C17—C22—C21	−52.3 (3)
C6—C7—C8—C14	166.19 (19)	C31—O5—C28—O4	−0.4 (4)
C26—C8—C9—C10	76.6 (2)	C31—O5—C28—C17	176.0 (2)
C7—C8—C9—C10	−44.8 (3)	C16—C17—C28—O4	−137.1 (2)
C14—C8—C9—C10	−162.80 (19)	C18—C17—C28—O4	−15.8 (3)
C8—C9—C10—C5	55.7 (3)	C22—C17—C28—O4	102.4 (3)
C6—C5—C10—C9	−65.2 (3)	C16—C17—C28—O5	46.6 (3)
C4—C5—C10—C9	160.1 (2)	C18—C17—C28—O5	167.90 (18)
C8—C7—C11—C12	45.3 (3)	C22—C17—C28—O5	−73.9 (2)
C6—C7—C11—C12	−179.96 (19)	C28—O5—C31—C32	−174.5 (2)
C7—C11—C12—O3	149.9 (2)	O5—C31—C32—C37	91.0 (3)
C7—C11—C12—C13	−35.4 (3)	O5—C31—C32—C33	−89.4 (3)
O3—C12—C13—C18	−8.7 (3)	C37—C32—C33—C34	−0.2 (4)
C11—C12—C13—C18	176.7 (2)	C31—C32—C33—C34	−179.9 (3)
O3—C12—C13—C14	−143.7 (2)	C32—C33—C34—C35	0.2 (4)
C11—C12—C13—C14	41.7 (3)	C33—C34—C35—C36	0.7 (5)
C12—C13—C14—C27	63.6 (2)	C34—C35—C36—C37	−1.5 (5)
C18—C13—C14—C27	−69.7 (2)	C33—C32—C37—C36	−0.5 (4)
C12—C13—C14—C15	−177.79 (19)	C31—C32—C37—C36	179.1 (3)
C18—C13—C14—C15	49.0 (3)	C35—C36—C37—C32	1.4 (5)
C12—C13—C14—C8	−57.4 (2)	C3—O1—C38—O2	−6.1 (3)
C18—C13—C14—C8	169.39 (18)	C3—O1—C38—C39	173.1 (2)
C9—C8—C14—C27	66.2 (2)		
